# Getting the full picture

**DOI:** 10.7554/eLife.46279

**Published:** 2019-03-26

**Authors:** Alessandro Luchetti, Ananya Chowdhury, Alcino J Silva

**Affiliations:** 1 Department of NeurobiologyUniversity of California Los AngelesLos AngelesUnited States; 2 Department of Psychiatry & Biobehavioral SciencesUniversity of California Los AngelesLos AngelesUnited States; 3 Department of PsychologyUniversity of California Los AngelesLos AngelesUnited States; 4 Integrative Center for Learning and MemoryUniversity of California Los AngelesLos AngelesUnited States; 5 Brain Research InstituteUniversity of California Los AngelesLos AngelesUnited States

**Keywords:** hippocampus, calcium imaging, acetylcholine, electrophysiology, freely behaving, sleep, Mouse

## Abstract

A combination of old and new techniques has revealed new details about the behavior of individual neurons across the sleep-wake-cycle.

**Related research article** Zhou H, Neville KR, Goldstein N, Kabu S, Kausar N, Ye R, Nguyen TT, Gelwan N, Hyman BT, Gomperts SN. 2019. Cholinergic modulation of hippocampal calcium activity across the sleep-wake cycle. *eLife* **8**:e39777. doi: 10.7554/eLife.39777

Electrodes have been used to excite and record electrical signals in the nervous system for decades. More recently other techniques, notably calcium imaging and optogenetics, have allowed neuroscientists to study a much wider range of phenomena in 'behaving' animals (i.e., animals that have not been anesthetized and are free to perform various tasks; Ziv et al., 2013; Kim et al., 2017; Stamatakis et al., 2018). Now, in eLife, Stephen Gomperts and colleagues at Massachusetts General Hospital – including Heng Zhou as first author – report how they have used a combination of electrode recordings and calcium imaging to study hippocampal neurons in mice engaged in learning and memory tasks (Zhou et al., 2019).

The hippocampus has a critical role in learning and memory. It is thought that signals sent along cholinergic neurons from other regions of the brain to the hippocampus place it in an 'information acquisition state' that is associated with the animal actively exploring its environment. These signals also modulate neuronal activity, including electrical excitations called theta oscillations, and the encoding of memory in the hippocampus (Hasselmo, 2006; Teles-Grilo Ruivo and Mellor, 2013). During theta oscillations, increases in the concentration of calcium ions can lead to changes in the strength of the synapses between neurons: this 'synaptic plasticity' is needed for the acquisition and storage of information in the hippocampus (Buzsáki, 2002). Later, a different hippocampal state, characterized by sharp waves and ripples (SWRs), is thought to replay and broadcast the information acquired to more permanent storage sites in the neocortex (Buzsáki, 2015). Unlike theta oscillations, which are generally smooth low-frequency waves, SWRs are more jagged and have higher frequencies; both single SWRs and trains of SWRs can be observed in the hippocampus.

To further study the cellular and circuit mechanisms engaged in these different functional states of the hippocampus, Zhou et al. used both electrode recordings and calcium imaging during the sleep-wake-cycle. As expected, the level of calcium activity in the hippocampus was highest during theta oscillations and lowest during sharp waves and ripples. Furthermore, although the timing of the calcium activity did synchronize with the ripples in SWRs, as previously thought (Malvache et al., 2016), the level of calcium activity only increased during trains of SWRs (and actually dropped during single SWRs). These are interesting results that will continue to fuel efforts to model the neural circuits in the hippocampus.

Zhou et al. also explored how calcium activity varied during the sleep cycle, and found that it was higher during rapid eye movement (REM) sleep than during slow wave sleep. Sleep is thought to have an important role in memory consolidation, with recently encoded memories being reactivated during slow wave sleep, and then consolidated during REM sleep (Sara, 2017). The latest findings suggest that different levels of calcium activity during the different phases of sleep could be key for memory consolidation.

Zhou et al. also studied how calcium activity was affected by theta oscillations and by signals sent along cholinergic neurons from the medial septum to the hippocampus. The cholinergic neurons were activated by expressing a chemogenetic receptor in the medial septum (Roth, 2016). This allowed the researchers to specifically activate the cholinergic neurons in the medial septum by administering the chemogenetic ligand to the animals. Activation of these neurons increased calcium activity in the hippocampus, but reduced sharp waves and ripples during periods of low mobility and sleep. Furthermore, using a drug to inhibit a subset of the receptors for cholinergic neurons reduced calcium activity while increasing sharp waves and ripples. The ability to record these two distinct signals, calcium activity and electrical oscillations and waves, revealed that calcium activity in the hippocampus depends on behavioral states, electrical activity, and cholinergic activation.

The work of Zhou et al. is representative of a novel wave of studies in systems neuroscience that is moving the field from its roots in electrophysiology to studies that involve recording and manipulating a wider range of biological phenomena. The present revolution in the field started more than 10 years ago with optogenetic tools capable of turning neurons on and off, and with neuronal imaging of 'head-fixed' animals (Kim et al., 2017; Stamatakis et al., 2018). It is now possible, as Zhou et al. have shown, to use head-mounted fluorescent mini-scopes to record cellular and circuit events in freely moving mice.

Moreover, in addition to allowing the activation and inactivation of specific neurons and neural circuits, a new generation of optogenetic and chemogenetic tools make it possible to manipulate specific molecular events in specific cells (not just neurons) in these circuits. These new tools are providing researchers with unprecedented access to functional states in circuits that had previously gone unrecognized. Zhou et al. have taken advantage of these advances to uncover a wealth of previously unknown interactions between phenomena traditionally probed in hippocampal research (i.e., electrophysiological states and cholinergic function) and phenomena revealed by the new techniques (i.e., calcium events) during a wide range of functional states, including quiet wakefulness, running, slow wave sleep and REM sleep. We can only wonder what we will continue to uncover as new tools allow us to dig deeper and see further into the functional complexity of brain circuits.
